# Supplementary material for: Splice-Junction-Based Mapping of Alternative Isoforms in the Human Proteome
Source: Cell Rep. Author manuscript; Available in PMC 2020 Jan 15. (PMC6961840; doi:10.1016/j.celrep.2019.11.026)
Supplement: 2 [file NIHMS1546469-supplement-2.zip › DF1/PXD006675/VentricularSeptum/VentricularSeptum_23_NEBL_TTQQNISAVK.pdf]

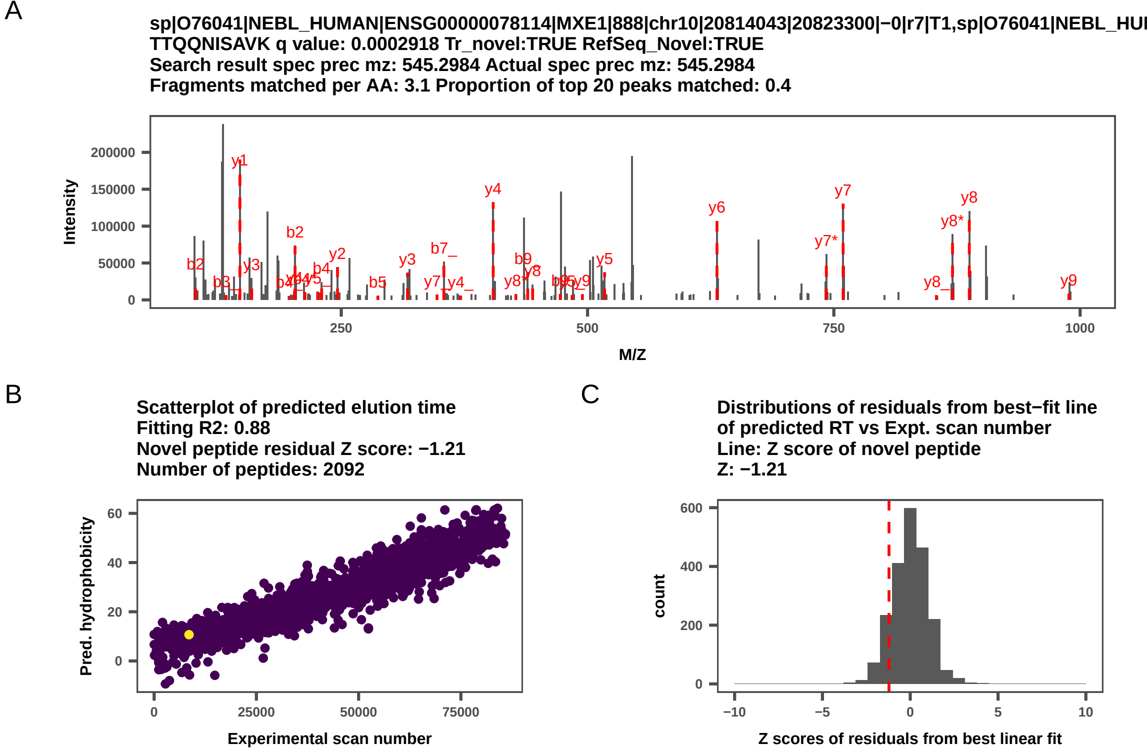
A
sp|O76041|NEBL_HUMAN|ENSG00000078114|MXE1|888|chr10|20814043|20823300|−0|r7|T1,sp|O76041|NEBL_HU
TTQQNISAVK q value: 0.0002918 Tr_novel:TRUE RefSeq_Novel:TRUE
Search result spec prec mz: 545.2984 Actual spec prec mz: 545.2984
Fragments matched per AA: 3.1 Proportion of top 20 peaks matched: 0.4
Intensity
200000
150000
100000
50000
0
250
500
750
1000
M/Z
y1
b2
y2
y3
y4
y5
y6
y7
y7*
y8
y8*
y9
b5
b9
B
Scatterplot of predicted elution time
Fitting R2: 0.88
Novel peptide residual Z score: −1.21
Number of peptides: 2092
Pred. hydrophobicity
60
40
20
0
25000
50000
75000
Experimental scan number
C
Distributions of residuals from best−fit line
of predicted RT vs Expt. scan number
Line: Z score of novel peptide
Z: −1.21
count
600
400
200
0
−10
−5
0
5
10
Z scores of residuals from best linear fit
